# Crash Narratives and Accidental Archives: Rethinking Road Safety in South Africa

**DOI:** 10.1080/03057070.2024.2513113

**Published:** 2025-06-11

**Authors:** Rebekah Lee

**Affiliations:** University of Oxford

**Keywords:** Road safety, road accident, South Africa, Road Accident Fund, accidental archive, road danger, crash narrative, road injury, body politics

## Abstract

This article considers the implications of historicising road safety in contemporary South Africa. It offers a critical interrogation of the ‘epidemiological turn’ evident in recent global road safety campaigns, primarily by ‘refiguring’ or decentring the archive on road safety in South Africa. Using the frame of ‘crash narratives’, this study assembles a diverse range of ‘fragments’ provided through oral testimonies and ethnographic observation of road users including township residents, transport operators, funeral entrepreneurs and their clients, as well as Road Accident Fund (RAF) claimants and associated medico-legal experts. Within this framing, stories of ‘black spots’ and ‘twice deaths’ – fatal road accidents en route to funerals – and the personal testimonies of RAF claimants offer a glimpse into wider historicised and contested dynamics around the nature and meaning of road danger and accidental death in South Africa. They also provide a more intimate window onto the lingering bodily and emotional trauma experienced by road accident victims, their families and caregivers, as well as suggest the contours of emergent domestic fault lines shaped by the politics of compensation engendered by the RAF system. Dwelling on this particular ‘accidental archive’ thus brings into sharper focus the lived experience of the road accident crisis in South Africa and, correspondingly, an appreciation of everyday forms of road safety already at work in township communities.

## Introduction

In early December 2022, at a time when South Africa was emerging from nearly two years of state restrictions on movement instigated by the Covid-19 pandemic, then-Minister of Transport Fikile Mbalula stood poised by a fairly nondescript but essential feature of road traffic regulation and control – a weighbridge – just off the busy national N3 highway on the outskirts of the historic Transvaal town of Heidelberg in what is now Guateng province. Flanked by officials from the Road Traffic Management Corporation (RTMC), the country’s main road safety authority, as well as the South African National Roads Agency (SANRAL), the parastatal charged with the development and maintenance of the national road network, Mbalula formally announced the launch of the newest iteration of the Arrive Alive campaign under the telling theme, ‘It Starts With You’. He urged road users to ‘exercise extreme caution and vigilance’ over the forthcoming festive season to ‘arrest the carnage on our roads’, asserting that ‘more than 90% of road traffic crashes can be directly attributed to the human element, which invariably relate to violation of traffic rules’. Tackling driver ‘ill-discipline’, as he termed it, was thus a matter of ‘collective responsibility’ that involved, primarily, concerted commitment on the part of individuals to curb irresponsible driving behaviours and the flouting of licensing and vehicle registration laws. Secondarily, collective responsibility required aggressive enforcement of these same laws by traffic and road authorities, a process Mbalula vividly described as ‘tighten[ing] the noose around the necks of those who continue to perpetuate unlawful conduct’. He reminded the audience of the sobering costs of failure: ‘[r]oad crashes resulting in serious injuries and fatalities continue to pose a huge burden to our economy, with a loss of more than R188 billion annually’. Mbalula resolutely concluded, ‘[t]his is a cancer we are determined to uproot if we are to make a telling difference in making our roads safer’.^1^

This article considers the implications of historicising road safety in contemporary South Africa. Despite the concerted efforts in recent years of state authorities as well as local, national and social media commentators to shine a spotlight on the manifold dangers inherent in South African roadscapes, the subject of road safety itself has received relatively little scholarly attention, with most existing research – like much of the global health-driven road safety scholarship – conducted firmly in the present tense. Although there is widespread consensus that road safety is a vital issue of concern across sub-Saharan Africa and integral to state developmentalist agendas, the conceptualisation of road safety is a fraught and contested terrain, constitutive of a broad array of competing interests, agents, forces and institutions at many different levels, both local and global. Rather than evoking a single set of universal truths, then, it is perhaps more helpful to look at the road safety sphere as a paradigm, as Thomas Kuhn would have it, a way of framing the world; and, like any other paradigm, subject to historical change.^2^

Take, for example, the Arrive Alive campaign’s explicit invocation of personal responsibility in curbing ‘risky’ behaviour – ‘It Starts With You’. In many respects, this slogan as well as Mbalula’s accompanying remarks at the Heidelberg weighbridge neatly encapsulate what Mark Lamont and I have termed the ‘epidemiological turn’ evident in recent African road safety initiatives.^3^ Road safety, in South Africa as elsewhere on the continent, has become firmly recast within the public health domain, distant and distinct from the technopolitics of road infrastructure, vehicular design and transport engineering. This epidemiological turn is evident in South Africa on highway billboards emblazoned with the cautionary words, ‘Speed Kills’, as well as in radio spots urging listeners to encourage their partners to ‘buckle up’, echoing the same discursive arc present in the HIV/AIDS ‘Be Wise/Condomise’ campaigns. Mbalula’s likening of the scourge of road accidents to a ‘cancer’ that needs to be excised has resonance with the medicalised framing adopted by his predecessor – for example, at the South African launch of the United Nation’s Decade of Action for Road Safety in 2011, then-Minister of Transport Sibusiso Ndebele famously likened the road safety predicament in South Africa to an ‘epidemic’ of crisis proportions and explicitly linked it to the country’s struggles amid the HIV/AIDS pandemic.^4^

To an extent, it could be argued that the turn to ‘personal responsibility’ in road safety is part of a global paradigm shift in road safety approaches and not specific to South African or even African contexts. As Peter Norton has shown in his concise history of traffic safety in the USA, four ‘approximately sequential paradigms’ can be identified, each with differently constructed notions of safety: ‘Safety First’, from the 1900s to 1920s, which cast speed as inherently negative and automobile drivers as innately ‘dangerous’; then ‘Control’, a technocratic focus between the 1930s and 1960s on safe road design, law enforcement and driver/pedestrian education; followed by ‘Crashworthiness’ in the 1970s, which focused on vehicular design and the use of driver restraints and airbags to engineer ‘safer’ accidents; and, finally, ‘Responsibility’ from the 1980s onwards, linked to increasing lay participation in road safety and influential campaigns such as Mothers Against Drunk Driving (MADD).^5^ However, an African trajectory of road safety, and arguably that of much of the global south, does not neatly map onto this sequential chronology of paradigms – for example, aspects of each of the four successive paradigms noted above can be discerned at work concurrently in contemporary African approaches to road safety.^6^

My research aims to explore how road safety in South Africa has been understood, constructed and practised in the *quotidienne*, and to examine the extent to which these more mundane frameworks and lived experiences of the nation’s road accident crisis coincide with public health positions on the same topic.^7^ Although the state’s evocation of road safety as a public health challenge has provided a recognisable script, particularly with respect to preventative strategies, the predominant stress on behavioural change in road safety campaigns has tended to obscure a broader consideration of systemic and historically contingent factors which have contributed to South Africa’s excessive road injury and fatality rates.^8^ This focus has also tended to ignore diverse local and culturally specific notions of risk and road danger, which I argue can exert a powerful influence on road users’ willingness to participate in accident prevention initiatives.^9^

This article casts a critical lens onto the epidemiological turn evident in South African road safety approaches by mapping everyday forms and meanings of road danger and road safety at work in urban township communities. Its analytic gaze lingers on and through a particular ‘alternative archive’ – what I tentatively call here ‘crash narratives’, which are narrative accounts of road injury or fatality – that emerged from oral history interviews and ethnographic observation of a diverse range of road users, including township residents, funeral entrepreneurs and their clients, transport operators and drivers, Forensic Pathology Officers (FPOs), as well as Road Accident Fund (RAF) claimants and associated medico-legal experts. Assembling crash narratives is a conscious attempt to engage in ‘refiguring’ or ‘decentring’ the archive on road safety, following Achille Mbembe’s invocation to search for ‘fragments’ of evidence of human experience beyond institutional repositories.^10^ By democratising the road safety archive to capture the experiences and processes of meaning-making of ordinary road users, this article opens up a more expansive set of alternative spatial, moral and political geographies suggested through these ‘new’ sources. As will be shown, crash narratives articulate novel conceptions of road danger, risky behaviour, accidental death and bodily harm, as well as their lingering traumas. They also suggest intriguing ways in which everyday forms of road safety and accident prevention have already been harnessed through conversation, ritual strategy and social practice.

## Routes through Road Safety Scholarship on Africa

Before illustrating the potential of crash narratives to illuminate the study of road safety in South Africa, it is useful to briefly sketch the wider scholarly and intellectual landscape of this field. The road safety sphere in sub-Saharan Africa is characterised by a general lack of intersectoral and inter-regional dialogue among various stakeholders, including government transport ministries, public health professionals and institutions, road engineers, automotive manufacturers, road safety advocates, transport operators, academic researchers and ordinary road users. This lack of dialogue is emulated in many of the internationally funded projects on road safety in the global south, particularly those with the stated aims to reduce road fatality and injury rates and to improve post-crash response.^11^

A critical and widely acknowledged weakness in the sub-Saharan African road safety sector is the lack of comprehensive and longitudinal data on many key indicators: numbers of road accidents, road fatalities and injuries (absolute and per crash), vehicle registration, unlicensed vehicles, driver licensing, and so on. Although South Africa is somewhat of an outlier in this regard due to the availability of robust and continuous domestic datasets dating back to the 1930s on these same indicators, blind spots remain – for example, the absence of racial classifications in much of the Transport Ministry’s data for the earlier periods means important shifts in vehicle ownership and registration among the black African population in the transitional and post-apartheid periods cannot be precisely located.^12^ The general lack of ‘baseline’ historic data in sub-Saharan African countries makes it difficult to meaningfully demonstrate or discern patterns of continuity or change, compounding problems in assessing the efficacy of targeted road safety interventions over time.^13^ Furthermore, in the main, the limited academic studies which do explicitly address road safety in the African context tend to exist in disciplinary siloes, with a decided lack of engagement particularly between the domains of medical and natural sciences on the one hand and the social sciences and humanities on the other.^14^ The creative arts tend to be excluded entirely from conversations on road safety, although this is beginning to be challenged.^15^

This more technocratic and health outcomes-orientated road safety scholarship is distinct and analytically separate from the vibrant historical and ethnographic research of the last three decades on African automobility, road ecosystems and livelihoods, and car cultures. Adeline Masquelier’s portrait of ‘road sirens’ haunting the highways of contemporary Niger and Luise White’s study of ‘vampire’ rumours in colonial east and central Africa implicating mobile bloodsucking police recruits, fire brigade officers and game rangers were two early seminal studies that vividly attested to the particular cosmological power and inherent anxieties Africans attached to roads as well as to the novel automotive technologies that plied them.^16^ Later studies expanded the geographical, affective and temporal trajectories of their work, such as key edited volumes on the history of motor vehicles in Africa, the ‘perils and possibilities’ of African roads, and the ‘making of’ the African road.^17^ Recently, there has been a veritable explosion of literature on emergent African automobilities that are marked by their careful historicisation and attention to socio-cultural dynamics, including Lindsay Green-Simm’s study of the cultural (re)production of automobility in west Africa, Kenda Mutongi’s nuanced social and economic history of *matatu* transport in Kenya, Jennifer Hart’s illuminating study of the ‘entrepreneurial mobility’ and ‘vernacular politics’ of Ghanaian drivers in 20th-century Accra, Joshua Grace’s sensitive portrayal of the ‘technopolitics’ of ruination and development within Tanzania’s transport and car repair industries, and most recently, Daniel Agbiboa’s compelling ethnographic meditation on precarity and corruption in Nigeria’s urban informal transport sector.^18^

Curiously, a geographical focus on southern Africa, and on South Africa specifically, is largely absent from this ethnographically rich and historically attuned scholarship on African roads and automobility, which has focused overwhelmingly on west and east African contexts. To a certain extent, South Africanist research has remained preoccupied with two dominant framings that have contributed to a more internalised and ‘exceptional’ focus at the expense of engagement with wider, transnational and transregional flows and discourses. The first framing, largely through the analytic lens afforded by human geography and the field of transport studies, contends with the mechanics and legacies of apartheid’s peculiar spatial engineering on South African transport and road infrastructure – see in particular Gordon Pirie’s manifold contributions to this rich literature.^19^ The long shadow cast by urban apartheid policy and planning measures can be seen, for example, in the axial nature of road networks in the post-apartheid city that continue to privilege connectivity of transport and commercial flows to and from urban centres, rather than across outlying areas. Its imprint can also be seen in township roadscapes which retain features once considered essential to the apartheid state’s mechanised vehicles of surveillance and control, with township boundaries defined by high-speed transport corridors and residential areas bisected by broad access roads, at the expense of sidewalks and other more pedestrian-friendly crossings, bypasses and intersections.^20^

The second scholarly preoccupation in South Africanist research concerns the violence and ‘big man’-ship associated with the minibus taxi industry.^21^ This research offers the potential for a salient comparative perspective on developments observed in west and east Africa, with seemingly striking parallels to *tro tro* drivers in Ghana and *danfo* minibus and *matutu* transport operators in Nigeria and Kenya, respectively, and may contribute to historically locating the emergence of a particular model of mobile entrepreneurism and the gendered construction of urban masculinities on the continent.^22^ However, by and large the orientation of research on the South African taxi industry remains inwardly focused on historicising and understanding the particular social and cultural roots of violent conflict between rival taxi operators and associations. Scant attention is paid to passenger perspectives and experiences. Although the subsequent social science literature on taxi ‘capitalisation’ and transport integration schemes has brought a wider lens onto analyses of state attempts to formalise, ‘modernise’ and regulate the taxi industry, this scholarship to an extent replicates rather than critically interrogates the predominant emphases on minibus owners and operators as the key arbiters, the industry’s overall violent orientation, and control as the central dynamic of concern in a fiercely competitive industry.^23^

Hedley Twidle’s experimental meditation on the national N2 highway is an intriguing exception to the trends in South Africanist scholarship noted above, as is Njogu Morgan’s study of urban mobility and bicycling in Johannesburg.^24^ Nonetheless, southern Africa remains a regional outlier in the recent burgeoning of Africanist scholarship on automobility and the technopolitics of the road. As yet, no major study in this vein on the South African context exists, despite the pivotal role of road infrastructure, the transport sector and the domestic automotive industry in the country’s political and economic development, as well as the centrality of cars and roadscapes in apartheid and post-apartheid literary and popular imaginaries.^25^

## Navigating Moral Geographies of the Road

Township funeral undertakers and their mobile clientele make for highly astute, if unlikely, commentators on the road accident crisis in contemporary South Africa. On Friday evenings a veritable convoy of minibus taxis forms along the N1 highway exiting Cape Town, bound for funerals taking place in the small towns and rural areas of the Eastern Cape. Speed is a valued commodity in the long-distance funeral trade, with taxi drivers urged on by grieving passengers and entrepreneurial bosses to ensure the punishing overnight journey (often between 1,000 and 1,200 kilometres) is completed on time.^26^ The stakes are even higher for those packed vehicles charged with transporting the body of the deceased, usually towed in a trailer behind. In an earlier study, I described the phenomena of ‘twice deaths’ or ‘double deaths’, which are fatal road accidents en route to a funeral, often involving the corpse in transit. Although the scale of twice deaths is difficult to quantify, apocryphal accounts of these events circulate widely and have become part of the parlance of township funeral cultures. Mourners utilise the telling of twice deaths as affective acts of moral reckoning; that is, as ways to bring to the surface contentious familial debates involving the deceased, whose active intervention in these debates is manifest through the occurrence of the accident itself. I have argued that stories of twice deaths have become potent repositories for the anxieties inherent in negotiating death over distance in post-apartheid South Africa.^27^ However, I have also come to realise that these accounts are not solely meditations on the uncertainties of death or the liminality of the deceased. They can alternatively be refigured as ‘crash narratives’, as evocative and instructive ways of thinking about road danger, the perils of speed, and the hazards of vehicular travel.

Responses to twice deaths can, correspondingly, be seen as a form of accident prevention. The central imperative of the deceased’s safe passage over distance is woven into the tight choreography of ritual traffic during a funeral weekend. A ‘sending-off’ ceremony on the Friday marks the departure of the deceased along with a cortege of mourners, with a brief formal ‘receiving’ of the coffin on the Saturday morning signalling the terminus of the corpse’s journeying from dense metropole to rural environs. At the funeral service later on the Saturday, a witness – usually a fellow passenger – verbally attests to the smooth transit of the deceased in a dedicated item on the service programme.^28^ Indeed, moral conduct towards the deceased-in-motion needs to be carefully scripted. This is particularly evident in a ritualised form of ‘talking to’ the dead in which a nominated kin member directly addresses the corpse at several key moments along its final journey, even including at rest stops on the road.^29^ At a sending-off ceremony in Crossroads township in Cape Town that preceded a 1,000-kilometre journey to the small town of Hofmeyr in the Eastern Cape, a male family member voiced this message to the deceased as the coffin was carried and carefully placed into the trailer: ‘[w]e are taking you from your sister because you came to Cape Town to get well, but it was not God’s will that you leave Cape Town breathing. We take you now, my cousin, and head towards Hofmeyr. Do not cause them hassle. Arrive safely in Hofmeyr because we cannot leave you here’.^30^ As this excerpt demonstrates, the stream of words directed at the deceased is remarkable for its prosaic force – seen in the admonishment, ‘Do not cause them hassle’ – as well as an acute sense of spatial positioning.^31^

Appeals for spiritual protection are not necessarily unique to South African roadscapes. In Niger, as Masquelier has shown, the use of protective amulets in vehicles and the scheduling of travel to avoid days deemed by diviners to be inauspicious or ‘heavy’ are local strategies to ward off potential bodily harm from vengeful ‘road sirens’ and other deadly spirits haunting Nigerien roads.^32^ Gabriel Klaeger similarly writes on the ‘everyday religiosity’ of road users in Ghana, observed through itinerant preachers on packed lorries, magical protections from witchcraft bought by drivers, routine prayers for safety alongside ritual blessings of vehicles before important journeys, and customised bumper stickers declaring God’s protection. Klaeger attests to the ‘intrinsic’ nature of these spiritual practices, how they are affective elements of ordinary Ghanaians’ being and traveling on the road.^33^ Yet, I contend that practices like ‘talking to’ the dead can also operate beyond their essential spiritual or affective dimension and are illustrative of a diverse repertoire of enacted measures that respond to the lived realities of road carnage. In that sense, verbally entreating the deceased to ‘behave’ en route while providing to the corpse vital, real-time co-ordinates of its direction of travel should be seen as attempts at safe navigation, an ‘everyday’ form of road safety charted through unpredictable and dangerous terrain.^34^

A key preoccupation of interlocuters when discussing these more everyday forms of road safety is how to confront the dilemma of recurring road accidents and fatalities. Twice deaths are but one manifestation of this fear. A single individual, over a lifetime, may accumulate multiple experiences of road injury and harm. A Western Cape-based neuropsychologist, who has conducted neurological assessments of claimants seeking compensation from the Road Accident Fund for their injuries, related the jarring moment she realised in the course of a clinical examination that one particular claimant, at the age of 32, had been the victim of his fourth road accident – all as a pedestrian. His first experience of road injury had been at four years of age. Certainly, this RAF claimant’s life experience is a stark reminder that pedestrians remain the most vulnerable of crash victims – in 2022, a staggering 43 per cent of the 12,436 people who died on South African roads were pedestrians.^35^ Despite the strictures of the RAF process, which limited clinical assessments to the most recent occurrence of road injury, the neuropsychologist was moved to reflect on the bodily and developmental harms such cumulative road traumas could wreak: ‘[y]ou look at someone [like him] and you think, he didn’t have a chance from the start’.^36^

Multiple crashes can also be *collectively* remembered within a family, as my interlocuters insist, like a slow-motion pile-up whose consequences reverberate beyond each affected individual or the temporal confines of an event. Take, for instance, the following account:

*Makhaya Mgangathi, an undertaker in Gugulethu township since 2002, was reflecting on the manifold road hazards encountered by funeral entrepreneurs when the conversation turned to a more personal sequence of events. He recalled the restless spirit of his great-great grandfather, who was violently killed at some point in the 1800s, most probably during the Xhosa frontier wars with British colonial forces in what is now Eastern Cape province. When I asked how his family knew that the spirit of his great-great grandfather was not at peace, he replied: [b]ecause of certain things that happened within our families. We are always involved in accidents. Not only most of the time, but seriously hurt or injured … I was involved in two car accidents, because of something that one of the drivers did … My father was held up at gunpoint, my brother was held up at gunpoint … I was held up at gunpoint, with two guns, and these guys they didn’t shoot but decided to come and kill me … And we said,* ‘*This is very strange’, because according to our culture it shouldn’t, certain things shouldn’t occur every now and again and it really raises suspicion.^37^*

*Members of Makhaya’s extended family separately consulted two* sangomas *(traditional healers), one in Cape Town and one in the Eastern Cape. He said of the* sangomas*’ diagnoses, ‘they told them exactly the same thing’, namely, that the spate of road accidents and violent events afflicting his family were the result of the great-great grandfather’s unnatural death and his burial in an unknown location. The only recourse would be to locate his bones – ‘Go and find his grave, to go and talk to him’ – and ceremonially re-inter his remains in the family’s ancestral burial ground. Makhaya admits that the search for the remains is ‘difficult’, particularly as his parents have passed away, taking with them valuable lineage and customary knowledge. ‘We haven’t found the spot, we need to’. But he remains undeterred, insisting that, ‘I’m not in a hurry’. What is more important is that the resolution be the correct one:* ‘*he* [the great-great grandfather] *still longs to be with his relatives, with his sons, grandsons, great-grandsons and so forth, you know … We are doing the right thing. You see, let me tell you this, for us as Xhosas it’s extremely important that we do the right thing once, the first time.*’^38^

I am interested in crash narratives like Makhaya’s for their potential to invite more historicised lines of questioning, particularly around the notion and framing of the ‘accidental’. The problem of accidental death and how to prevent its recurrence is certainly not a new feature of Xhosa cosmology or social institutions.^39^ Although the intellectual, political and legal administrative trajectories of the accidental have been effectively mapped and problematised in the global north, as Judith Green’s seminal study illustrates, no comparable project exists in the South African or wider sub-Saharan African historical context.^40^ As Makhaya’s telling search for his great-great grandfather’s remains suggests, crash narratives tend not to be expressed solely within the immediate time frame bounded by the accident itself. Often embedded in these collective stories is a longer historical arc, a framing that links together the crash with a more expansive accounting of occurrences, forces and choices.

Like Makhaya, other respondents spontaneously recalled earlier personal and familial experiences of road injury and death by way of prefacing the accident in question, as well as connecting road accidents to other forms of unnatural deaths, including violent death.^41^ That an individual experience of a road accident could be incorporated into a larger collective narrative – ‘we are always involved in accidents’ – is underlined in Makhaya’s account and resonates with how many such ‘accidental’ testimonies are framed as a search for cumulative meaning. Indeed, gatherings of extended kin at occasions such as funerals and during the Easter and Christmas holiday periods have become moments when such communal reckoning can occur, although respondents attest these gatherings are often characterised by uncertainty over chronologies – how far back can one begin to date collective misfortune? – and intense disputes over the ‘proper’ manner of a dilemma’s resolution.^42^ Yet, effective relief, while elusive, remains a hoped-for prospect. Transport activist Welcome Malahle described a spate of road accidents which struck his family with alarming frequency, repeatedly in November, December and January ‘each and every year’, until a lasting resolution was found by re-interring the remains of an estranged family member:

[We] gathered together and we went to the grave of our ancestor … [We asked], ‘Is there anyone who has a solution on this issue?’ Some said maybe Mpokela is upset with what we are doing, then we dig his grave and rebury him again. Same day, same spot and same grave, we just bought a new blanket. It was no longer bones, it was something that shows that there were bones here. We took the soil and put it in the blanket and buried him again. Then we slaughter[ed] a sheep after that. We slaughter[ed] a cow, then we made *umqombothi* [a traditional South African beer] and after that, the accidents were gone. From 2006, we never had anyone who had an accident in my family.^43^

That recurring road accidents have a particular *spatial* dimension was another common refrain in these testimonies. During a wide-ranging discussion with two minibus taxi operators in Khayelitsha township about the travails of taxi ownership in 21st-century South Africa, the conversation turned to a certain notoriously dangerous section of the route to the Eastern Cape. As Khwezi Buyani described it:

The long distance … the road to Eastern Cape, it’s difficult because it has got trucks and after Beaufort West to Grahamstown it’s narrow and there’s no lights. On the road, it’s dark. The spirits of those who died on the road, and their families [who] have never come to fetch the spirit, they also contribute to the accidents.^44^

Welcome Malahle similarly identified an adjacent treacherous stretch of road, a high-speed corridor on the N1 national highway, on the same route to the Eastern Cape:

I’m sure the number of people who are causing these road deaths between Laingsburg and Beaufort West are the people who were Christians because they [the spirits] were left there … As cultural custodians, we believe that we must go there and pick that spirit, so that we can go and bury that person. [Otherwise,] those accidents will keep and keep on till they fix that problem.^45^

Rumours of these so-called ‘black spots’ circulate widely and serve as precautionary locational pointers of hazardous road conditions that should be avoided, particularly at night, or undertaken with great vigilance.^46^

These narratives are not confined to well-plied transit routes in South Africa. Indeed, an alternative mapping of southern African roads comes into view when stitching together popular and media accounts of black spots in the wider region. According to journalist Robyn Dixon, in Zimbabwe a section of the road extending south of Harare, known as ‘52’ for the relevant kilometre marker, became a particularly potent and feared location after multiple crashes occurred there in 2009, including an incident which took the life of Susan Tsvangirai, the wife of then-Prime Minister Morgan Tsvangirai.^47^ In a detailed section of a paper on ‘explanations’ of road accidents in Zambia, scholar Webby Kalikiti meticulously specified the following black spots to avoid in Zambia, comprised of vital co-ordinates ‘picked out’ by his informants:

Kapiri Ngozi along the Lusaka Chirundu road, the area around Shimabala on the Lusaka Kafue road, Landless corner on the Lusaka Kabwe road, a stretch on the same road near Zambia National Service, a corner near Mulungushi Textiles in Kabwe, the area between Chainanama and Mumana Pleasure resort in Lusaka and Mubanga-Chipoya Basic School in Kasama.^48^

Importantly, however, as Khwezi’s and Welcome’s comments suggest, the naming of black spots is not a value-neutral demarcation of high-accident zones, but a spatially fixed attribution of moral responsibility. When the surviving family members of a road fatality fail to retrieve the deceased person’s spirit (*umphefumlo* in Xhosa, also meaning ‘breath’ or soul) from the scene of a road accident, that spirit becomes unstable, increasing the risk for another accident in the same location. Isak Niehaus observed a similar dynamic at work in Bushbuckridge, where local residents ascribed the recurrence of road accidents at a given location to the unappeased breath or *moya* of a deceased person that remained at the scene.^49^ Interestingly, however, while villagers in the Lowveld resorted to the services of a diviner to contain *moya* and return it to a safer destination, my interlocuters insisted the primary duty in ritually cleansing a black spot remained with the family members of the deceased. As Makhaya explained:

[Family members] need to come and make sure that they also look, look for a small stain and make sure that stain is removed … you must take that person’s soul from the place … We talk to you [the deceased] and say, ‘Look, this is where you died, and we want to make sure that … you don’t go to that spot, instead to go to your house, to your family, to a place where you know where your family is’.^50^

Christians were particularly derelict in this shared responsibility, as seen in Welcome’s comments above. This sentiment was echoed by Khwezi:

These church people, who say they are ‘born again’, they are the problematic ones because they don’t believe in that. They never go back to the accident scene to fetch the spirits of their dead family members … They don’t pay attention to those things even though they are affecting other people.^51^

Welcome attested to the persistence of these forms of what he called ‘community practices’ around road accident sites and acknowledged his own family’s continuing participation in such methods of redress. But he admitted that in his work as a transport activist for the Public Transport Voice, a nascent commuter advocacy group based in Khayelitsha, little space was afforded to publicly divulge and describe such practices for fear of being discredited – ‘they will label me’, he insisted. He likened this suppression to his experience of the sidelining of meaningful dialogue around traditional medicine and healing in HIV/AIDS advocacy circles, a space he knew intimately as a former Treatment Action Campaign (TAC) employee. Yet, despite their apparent ‘invisibilisation’ in the public sphere, these alternative ways of knowing and speaking about road danger emerge as a resilient register, among several, employed by Welcome and other interlocuters.^52^

Indeed, respondents demonstrated an adept ability to traverse multiple discursive registers in our conversations, including those that more closely aligned with the state’s behaviourist approach to road safety. For example, while decrying the negligence of ‘born-agains’ in tending to black spots on the road, the taxi operators were also at pains to point out their own taxi association’s recent efforts to curb poor driving behaviours within its ranks – including fining taxi drivers for drinking before their shifts, on an ever-increasing scale of penalties – as demonstrations of their faithful adherence to road safety conventions. They also countered that such internally derived mechanisms of surveillance and control were ultimately ineffective without firmer policing of the behaviour of road safety authorities themselves, citing in particular the corrupt conduct of traffic officials.^53^ In this view, their transport businesses were effectively held ransom by traffic officers stationed at busy township thoroughfares, who issued fines to passing minibus taxis irrespective of whether or not a traffic violation had been committed. Certainly, respondents voiced a profound lack of confidence in the measures, processes and structures that together comprise the public arm of road safety management and enforcement in South Africa, from the issuance of driving licences and vehicle registration, to vehicular roadworthiness and speed compliance, the construction and maintenance of safe crossing zones for pedestrians and children in high-accident areas, and police and emergency response times for road accidents, particularly those occurring in townships.^54^ Thus, responses to black spots and twice deaths need to be seen as existing alongside, rather than wholly displacing, other ways of understanding and critiquing the technopolitics of road accident and injury in South Africa.

## The Road Accident Fund and the Intimate Body Politics of Road Injury

I conclude by briefly sketching the contours of a different moral economy of road accident and injury, as suggested in Apiwe Ginya’s account:

*In 2008, Apiwe’s mother died at the scene of an accident at the main bus terminal in central Cape Town, struck while caught between the paths of two buses. Apiwe was seven at the time. Apiwe was subsequently raised by his grandmother and then, after her passing in 2011, his maternal uncle. Apiwe’s father remained throughout his childhood as only a passing presence. At 14 years of age, Apiwe’s uncle informed him that, as a surviving child of a fatal road accident victim, he would be entitled to a lump sum cash payout from the RAF when he turned 18. Apiwe remembered that at the time, he did not dwell on this revelation nor on the RAF, not wanting to believe in ‘something that I’m not quite sure of’. Apiwe’s older sister received her payout before he did, and although at the time he did not know the specifics regarding the amount of her entitlement nor her subsequent expenditures, he did notice ‘she started partying’ and was able to purchase a new car.*

*As Apiwe was approaching his 18th birthday, his father reappeared in his life and they spent considerable time together. Apiwe remembered his father’s generosity in those moments, demonstrable acts such as when the father covered his college fees for two months. In the days leading up to his birthday, his father helped Apiwe register for his bank card with ABSA Bank. According to Apiwe, on the day of his 18th birthday he received his R70,000 payout from the RAF. His father, armed with the ABSA card as well as the PIN he had taken note of earlier when helping Apiwe set up his account, promptly withdrew half of the payout and, without delay, bought a Toyota car with the funds. Apiwe attempted to contact his father, to no avail. He then began proceedings against his father at Khayelitsha Magisterial Court, but the court was unable to serve the necessary legal papers to return the funds because the father did not have a fixed abode. Apiwe had intended to use his RAF payout to financially assist his maternal uncle, to renovate what was intended to be their future shared abode. The week before our interview, his uncle was shot to death, in what Apiwe believed was a case of mistaken identity.^55^*

The RAF looms large in crash narratives such as Apiwe’s. Given the vast financial scale of RAF’s operations – with payment claims made in the 2019/20 financial year amounting to R39.5 billion and compensation paid out totalling R29.8 billion – the academic research on the RAF is, surprisingly, insubstantial. Notably absent from existing scholarship on the RAF is attention to the perspectives and experiences of road accident victims and their surviving carers and family members, with much of the social scientific literature instead focused on understanding evolving legislation and the role of lawyers, doctors and ministerial and administrative staff within RAF’s complex procedural framework.^56^

Signed into law by then-President Nelson Mandela in late 1996 and promulgated in May 1997, the Road Accident Fund Act established a national compensatory system which provided for medical expenses and loss-of-income support for road accident victims, from the date of the accident into the future, and also for funeral expenses and other forms of support for fatal road accident victims’ surviving families.^57^ According to its official website, the RAF:

provides compulsory cover to all users of South African roads, citizens and foreigners, against injuries sustained or death arising from accidents involving motor vehicles within the borders of South Africa. This cover is in the form of indemnity insurance to persons who cause the accident, as well as personal injury and death insurance to victims of motor vehicle accidents, and their families.^58^

The RAF was, and remains, funded by a nationwide levy placed on fuel.^59^ From its inception, the RAF has been plagued by financial difficulties, partly due to legal transaction costs and the unsustainably high levels of compensation paid out to victims. Beginning in 1999, Judge Kathy Satchwell chaired a multi-year review into the RAF’s finances and procedures. Following the report of the Satchwell Commission in 2002, some of its recommendations were integrated into the Road Accident Fund Amendment Act of 2005, including limiting RAF’s liability for compensation on general damages claims to cases of ‘serious injury’ only, and reducing the threshold of compensation on claims for loss of income.^60^

I am interested in the RAF as an underexplored yet essential feature of post-apartheid South Africa’s ‘redistributional regime’.^61^ Emergent research on the politics and mechanics of state redistribution has focused on its inherent extractive character. For example, Erin Torkelson has highlighted how the electronic cash payment system underpinning the state’s social assistance programme has provided fertile ground for the egregious harnessing of predatory financial instruments which, effectively, have increased the indebtedness of social service grantees rather than ensured their sustenance or social mobility.^62^ In a similar vein, I have begun to research the predatory infrastructures and accounting processes that have become emblematic features of the RAF system, particularly those associated with legal advice to, and medical assessments of, RAF claimants.^63^

However, for the purposes of this article, I turn instead to considering RAF payouts as a site of contention – and even predation – *within* households, as Apiwe’s narrative poignantly illustrates. This emphasis is part of a sustained seam of scholarship which has examined the intimate dynamics and domestic politics of various forms of welfarist provision in the transitional and post-apartheid periods, including old-age pensions, disability and child support. This research has highlighted in particular the gendered and generational fault lines generated by unequal access to these redistributive payments and systems.^64^ Observing the impact of RAF payouts on familial dynamics may likewise offer a potential window onto evolving, and deeply contested, relations of reciprocity within South African households. For Apiwe, the anticipated RAF payout was meant to signal his coming of age in multiple respects, not least as a significant financial contributor to the property he intended to renovate and share with his uncle. Instead, the occasion of his RAF disbursement was indelibly marked by the experience of paternal betrayal, on the one hand, and the eventual loss of his primary guardian on the other.

Furthermore, Apiwe’s failed attempt to pursue his father through the local magisterial court suggests the difficulties faced by RAF beneficiaries ‘cheated’ of their payouts who then seek legal redress against family members.^65^ Indeed, the very defining of ‘family’ can become enmeshed in complex legal-administrative accounting exercises and weighted with fiscal as well as moral significance, as Maxim Bolt has shown in his research on disputes over home ownership in Johannesburg townships.^66^ Proving close kinship appears to be an essential, yet fraught, part of the RAF claims process, particularly in cases of fatal road accidents. Sharing the same surname of a fatal road accident victim, for example, may offer a more straightforward route to successfully navigate RAF claims administration as a ‘surviving family’ member, regardless of that person’s caring relation to the victim. This may take away the opportunity for other kin to make bona fide claims on the RAF process.^67^ This is suggestive, rather than conclusive, of how the very terms and boundaries of ‘family’ may be utilised in, and also re-configured through, the bureaucratic administration and financial dispensation afforded via the RAF process.

## Conclusion

This article presents an initial attempt at refiguring and decentring the archive on road safety in South Africa, using the frame of crash narratives as an analytical route through a diverse range of fragments provided through oral testimonies and ethnographic observation. Within this framing, stories of black spots, twice deaths and the personal testimonies of RAF claimants have offered a glimpse into wider historicised and contested dynamics around the nature and meaning of road danger and accidental death as well as a window onto the intimate politics of compensation and redistribution in the post-apartheid period.

Crash narratives also reveal an emergent local repertoire of road safety practice. Cleansing black spots, re-interring the remains of the dead, and ensuring the integrity of the deceased-in-transit are not simply symbolic affects of, or discursive allusions to, the precarity of life on South African roads. These are also mobile technologies which are enacted and performed, and have lived effects.^68^ Rather than viewing the contemporary road safety crisis as predominantly a public health challenge, acknowledging it as a potential moral and ethical dilemma thus opens up for consideration popular understandings of road danger as well an appreciation of everyday forms of road safety already at work.^69^


Rebekah Lee *African Studies Centre, Oxford School of Global and Area Studies, University of Oxford, 13 Bevington Road, Oxford, OX2 6LH, United Kingdom. Email: rebekah.lee@africa.ox.ac.uk*
https://orcid.org/0000-0003-3368-3971


## Footnotes

1 South African Government, ‘Minister Fikile Mbalula: 2022 Festive Season Road Safety Campaign’, 8 December 2022, available at https://www.gov.za/news/speeches/minister-fikile-mbalula-2022-festive-season-road-safety-campaign-08-dec-2022, retrieved 30 December 2023.

2 P. Norton, ‘Four Paradigms: Traffic Safety in the Twentieth-Century United States’, *Technology and Culture*, 56, 2 (2015), pp. 319–34; T. Kuhn, *The Structure of Scientific Revolutions* (Chicago, University of Chicago Press, 1962).

3 M. Lamont and R. Lee, ‘Arrive Alive: Road Safety in Kenya and South Africa’, *Technology and Culture*, 56, 2 (2015), pp. 464–88.

4 In 2020 the United Nations resolved to renew this commitment and launched, with the World Health Organization, the second global UN Decade of Action for Road Safety, 2021–30, of which South Africa remains a committed partner: World Health Organization, ‘Decade of Action for Road Safety, 2021–2030’, available at https://www.who.int/teams/social-determinants-of-health/safety-and-mobility/decade-of-action-for-road-safety-2021-2030, retrieved 30 June 2023. South African Government, ‘Address by Sibusiso Ndebele at the Launch of the “Decade for Action for Road Safety 2011–2020”’, 11 May 2011, available at https://www.gov.za/news/speeches/address-launch-decade-action-road-safety-2011-2020-mr-sibusiso-ndebele-mp-minister, retrieved 2 June 2011. For a critique of the framing of the African road safety crisis as ‘epidemic’, see M. Lamont, ‘An Epidemic on Wheels? Road Safety, Public Health and Injury Politics in Africa’, *Anthropology Today*, 26, 5 (2011), pp. 3–5.

5 Norton, ‘Four Paradigms’; for an alternative periodisation of US road safety history, see C. MacLennan, ‘From Accident to Crash: The Auto-Industry and the Politics of Injury’, *Medical Anthropology Quarterly*, 2, 3 (1998), pp. 233–50.

6 Indeed, we argue that the history of road safety in sub-Saharan Africa, paradigmatic or otherwise, has yet to be comprehensively mapped: Lamont and Lee, ‘Arrive Alive’.

7 The research for this article is derived from two ongoing projects, with research primarily based in the Western Cape in the historically black African townships of Cape Town and focused on the Xhosa-speaking population, as well as secondary field sites in East London and the rural areas of the Eastern Cape. The first body of interview material and ethnographic research for this article was derived from a long-standing study on the changing meaning and management of death in transitional and post-apartheid South Africa. This was originally part of a larger collaborative project with Megan Vaughan on death in African history, funded by the Arts Humanities Research Council (UK). I am currently completing the monograph, entitled *Death and Memory in Modern South Africa*, based on this research. Second, I undertook fieldwork in 2016–17 and in 2019 with seed funding from the Wellcome Trust for the project, ‘Why do road safety interventions in South Africa fail? Using cultural and historical perspectives to solve a public health dilemma’. This research entailed scoping potential archives, conducting interviews and focus group discussions with a range of road users and road safety stakeholders, and further ethnographic observation and gathering of oral histories.

8 Lamont and Lee, ‘Arrive Alive’; R.C. Morris, ‘Accidental Histories, Post-Historical Practice? Re-Reading Body of Power, Spirit of Resistance in the Actuarial Age’, *Anthropological Quarterly*, 83, 3 (Summer 2010), pp. 581–624.

9 On local understandings of road danger in Africa, see, for example: A. Masquelier, ‘Road Mythographies: Space, Mobility, and the Historical Imagination in Postcolonial Niger’, *American Ethnologist*, 29, 4 (2002), pp. 829–56; G. Klaeger, ‘Stories of the Road: Perceptions of Power, Progress and Perils on the Accra-Kumasi Road, Ghana’, in K. Beck, G. Klaeger and M. Stasik (eds), *The Making of the African Road* (Leiden, Brill, 2017), pp. 86–115; M. Lamont, ‘Accidents Have No Cure! Road Death as Industrial Catastrophe in Eastern Africa’, *African Studies*, 71, 2 (2012), pp. 174–94.

10 A. Mbembe, ‘The Power of the Archive and its Limits’, in C. Hamilton, V. Harris, J. Taylor, M. Pickover, G. Reid and R. Saleh (eds), *Refiguring the Archive* (Dordrecht, Kluwer Academic Publishers, 2002), pp. 19–26; A. Mbembe, ‘Decolonizing Knowledge and the Question of the Archive’, public lecture at the Wits Institute for Social and Economic Research (WISER), University of the Witwatersrand (Johannesburg, 2015), available at https://wiser.wits.ac.za/system/files/Achille%20Mbembe%20-%20Decolonizing%20Knowledge%20and%20the%20Question%20of%20the%20Archive.pdf, retrieved 2 February 2023.

11 During this research period, I was involved in the evaluation for major grant funding of collaborative projects involving transnational consortiums working on improving African road safety, variously conceived. It was illuminating to observe which projects were viewed as ‘valuable’ and inherently ‘fundable’ in the road safety arena. Many proposals were highly specialised, employing advanced industrial engineering techniques, specialist technical equipment, software and expertise, with only a cursory nod to historical, social and psychological dimensions of road injuries, fatalities and post-accident trauma.

12 Morris, ‘Accidental Histories’, p. 597.

13 The ‘problem of the baseline’ is a key challenge more generally for historians of health in Africa and affects the accurate assessment of the efficacy of many health and development interventions in the post-colonial context. See R. Lee, *Health, Healing and Illness in African History* (London, Bloomsbury, 2021).

14 For an example of a ‘classic’ epidemiological approach to African road safety, see W. Odero, ‘Road Traffic Accidents in Kenya: An Epidemiological Appraisal’, *East African Medical Journal*, 72, 5 (1995), pp. 299–305. In contrast, Karl Peltzer and Walter Renner offer a novel, if exceptional, survey-based psycho-social analysis of wellbeing post-crash: K. Peltzer and W. Renner, ‘Psychosocial Correlates of the Impact of Road Traffic Accidents Among South African Drivers and Passengers’, *Accident Analysis and Prevention*, 36, 3 (2004), pp. 367–74. Mitchel Chatukuta and colleagues’ more recent interdisciplinary epidemiological study similarly looks at psycho-social perspectives but in the Namibian road safety context: M. Chatukuta, N. Groce, J. Mindell and M. Kett, ‘The Availability of Psychological Support Following Road Travel Injuries in Namibia: A Qualitative Study’, *PLos ONE*, 16, 10 (2021).

15 The potential role of the creative arts in both the production of knowledge on African road safety as well as in developing road safety interventions was a prominent question discussed with participants in the Radcliffe Institute (Harvard University) Exploratory Seminar on Road Safety in Africa that I convened in June 2022.

16 L. White, ‘Cars Out of Place: Vampires, Technology and Labor in East and Central Africa’, *Representations*, 43 (1993), pp. 27–50; A. Masquelier, ‘Encounter with a Road Siren: Machines, Bodies and Commodities in the Imagination of a Mawri Healer’, *Visual Anthropology Review*, 8, 1 (1992), pp. 56–69.

17 J. Gewald, S. Luning and K. van Walraven (eds), *The Speed of Change: Motor Vehicles and People in Africa*, *1890–2000* (Leiden, Brill, 2009); G. Klaeger (ed.), Special Issue on Ethnographies of the Road, *Africa: Journal of the International African Institute*, 83 (2013); K. Beck, G. Klaeger and M. Stasik (eds), *The Making of the African Road* (Leiden, Brill, 2017).

18 K. Mutongi, *Matutu: A History of Popular Transportation in Nairobi* (Chicago, Chicago University Press, 2017); J. Hart, *Ghana on the Go: African Mobility in the Age of Motor Transportation* (Bloomington, Indiana University Press, 2016); J. Grace, *African Motors: Technology, Gender, and the History of Development* (Chapel Hill, Duke University Press, 2021); D.E. Agbiboa, *They Eat Our Sweat: Transport Labor, Corruption, and Everyday Survival in Urban Nigeria* (Oxford, Oxford University Press, 2022); L.B. Green-Simms, *Postcolonial Automobility: Car Culture in West Africa* (Minneapolis, University of Minnesota Press, 2017). See also M. Rizzo, *Taken for a Ride: Grounding Neoliberalism, Precarious Labour, and Public Transport in an African Metropolis* (Oxford, Oxford University Press, 2017). For a useful examination of transport provision and development dynamics in the wider global south, see D. Simon, *Transport and Development in the Third World* (London, Routledge, 1996).

19 See, for example, G.H. Pirie, ‘Traveling under Apartheid’, in D.M. Smith (ed.), *The Apartheid City and Beyond: Urbanisation and Social Change in South Africa* (London, Routledge, 1992), pp. 173–82; G.H. Pirie, ‘Johannesburg Transport, 1905–1945: African Capitulation and Resistance’, *Journal of Historical Geography*, 12, 1 (1986), pp. 41–55; G.H. Pirie, ‘Implanting Racial Ideology: Bus Apartheid in Cape Town’, *Social Dynamics*, 15, 1 (1989), pp. 61–74.

20 A.P. Czeglédy, ‘Getting Around Town: Transportation and the Built Environment in Post-Apartheid South Africa’, *City & Society*, 16, 2 (December 2004), pp. 63–92; D. Japha and M. Huchzermeyer, ‘The History of the Development of Townships in Cape Town, 1920–1992’, *The Integration and Urbanisation of Existing Townships in South Africa Working Paper No. 2* (Newcastle, University of Newcastle, and Cape Town, University of Cape Town, 1995).

21 For example, see the following article-length treatments: M. Khosa, ‘Routes, Ranks and Rebels: Feuding in the Taxi Revolution’, *Journal of Southern African Studies*, 18, 1 (1992), pp. 232–51; E. Bahre, ‘A Trickle-Up Economy: Mutuality, Freedom and Violence in Cape Town’s Taxi Associations’, *Africa: The Journal of the International African Institute*, 84, 4 (2014), pp. 576–94; T. Gibbs, ‘Becoming a “Big Man” in Neo-Liberal South Africa: Masculinities in the Minibus-Taxi Industry’, *African Affairs*, 113, 452 (2014), pp. 431–48; L.J. Bank, ‘The Making of the Qwa Qwa “Mafia”? Patronage and Protection in the Migrant Taxi Business’, *African Studies*, 49, 1 (1990), pp. 71–93.

22 See Hart, *Ghana on the Go*; Mutongi, *Matatu*; Agbiboa, *They Eat Our Sweat*.

23 On reform in the minibus taxi industry see, for example, A H. Schalekamp and R. Behrens, ‘Engaging Paratransit on Public Transport Reform Initiatives in South Africa: A Critique of Policy and an Investigation of Appropriate Engagement Approaches’, *Research in Transport Economics*, 29, 1 (2010), pp. 371–8; J. Walters, ‘Overview of Public Transport Policy Developments in South Africa’, *Research in Transport Economics*, 22, 1 (2008), pp. 98–108; J. Barrett, ‘Organizing in the Informal Economy: A Case Study of the Minibus Taxi Industry in South Africa’, *SEED Working Paper No. 39* (Geneva, International Labour Organization, 2003).

24 H. Twidle, ‘N2: Reading, Writing, Walking the South African Highway’, *Social Dynamics*, 43, 1 (2017), pp. 59–81; N. Morgan, ‘Cycling Infrastructure and the Development of a Bicycle Commuting Socio-Technical System: The Case of Johannesburg’, *Applied Mobilities*, 4, 1 (2019), pp. 106–23.

25 See recent evocative literary treatments of taxi and road violence in novel and memoir form by Thabo Jijana, Imraan Coovadia and Sifiso Mzobe, as well as older expressions of township roadscapes by Es’kia Mphahlele: T. Jijana, *Nobody’s Business: A Memoir* (Johannesburg, Jacana, 2014); I. Coovadia, *The Institute for Taxi Poetry* (Cape Town, Random House Struik, 2012); S. Mzobe, *Young Blood* (Cape Town, Kwela, 2010); Es’kia Mphahlele, *Down Second Avenue* (London, Faber & Faber, 1959). Academic studies of this literature include M. Wessels, ‘Sifiso Mzobe’s *Young Blood*: Spaces of Getting and Becoming in Post-Apartheid Durban’, *Scrutiny*, 21, 1 (2016), pp. 87–101; M. Jones, ‘Conspicuous Destruction, Aspiration and Motion in the South African Township’, *Safundi*, 14, 2 (2013), pp. 209–24.

26 Interview with Mthetho Sibaya, 25 July 2017; interview with Sebonga Vetbooi, 6 August 2008; interview with Mphumelelo Mninzi, 6 October 2008; interview with Bekisa Dwangu, 27 November 2008; interview with Makhaya Mgangathi, 1 December 2008.

27 R. Lee, ‘Death in Slow Motion: Funerals, Ritual Practice and Road Danger in South Africa’, *African Studies*, 71, 2 (2012), pp. 195–211.

28 Interview with Vuyiseka Mfundisi, 18 September 2008.

29 Interview with Linda Gcina, 4 December 2008; interview with Bekithemba Mbekeni, 17 August 2017; interview with Bekisa Dwangu, 27 November 2008.

30 This particular sending-off ceremony and other aspects of the ritual traffic from Cape Town to the Eastern Cape are documented in my ethnographic film on the township funeral industry, ‘The Price of Death’ (2012).

31 I have argued elsewhere that this ‘talking to’ the corpse is an emergent form of ‘anti-technology’ which deliberately slows down the mobile trajectory of the dead, a way of reversing the spiritual disorientation of the deceased. See Lee, ‘Death in Slow Motion’.

32 Masquelier, ‘Road Mythographies’, p. 844–5.

33 G. Klaeger, ‘Religion on the Road: The Spiritual Experience of Road Travel in Ghana’, in J. Gewald, S. Luning and K. van Walraven (eds), *The Speed of Change: Motor Vehicles and People in Africa*, 1890–2000 (Leiden, Brill, 2009), pp. 212–31.

34 Interview with Vuyiseka Mfundisi, 18 September 2008.

35 Road Traffic Management Corporation (RTMC), ‘State of Road Safety in South Africa (January 2022 to December 2022)’, 2023, available at https://www.rtmc.co.za/images/rtmc/docs/traffic_reports/calendar/2022-State_of_Road_Safety_Report.pdf, retrieved 12 May 2025.

36 Interview with Katie Hamilton, 21 July 2017.

37 Interview with Makhaya Mgangathi, 1 December 2008.

38 *Ibid*.

39 Missionary and travellers’ accounts as well as early ethnographic sources provide some evidence of 19th-century Xhosa responses to deaths from unnatural causes such as by lightning strikes and drowning. John Henderson Soga, in his interwar auto-ethnography of the Xhosa, relates how burial practices were adapted in cases of unnatural death. Interring the deceased with their back facing the familial homestead was meant to prevent the same manner of death from striking the family again. J.H. Soga, *The Ama-Xosa: Life and Customs* (Lovedale, Lovedale Press, 1931). For a more recent evocation of indigenous theorisings on death, see Hugo Ka Canham’s important work on Mpondoland: H. Ka Canham, *Riotous Deathscapes* (London, Duke University Press, 2023).

40 J. Green, *Risk and Misfortune: The Social Construction of Accidents* (London, Routledge, 1997).

41 Interview with Welcome Malahle, 12 July 2017; interview with Minah Koela, 3 July 2017; interview with Malixole Raxho, 2 November 2008.

42 See H. Whyte, ‘Outside the Dwelling of Culture: Estrangement and Difference in Postcolonial Zululand’, *Anthropological Quarterly*, 83, 3 (2010), pp. 497–518; Ka Canham, *Riotous Deathscapes*.

43 Interview with Welcome Malahle, 12 July 2017.

44 Interview with Khwezi Buyani and Babalo Ncwadi, 20 July 2017.

45 Interview with Welcome Malahle, 12 July 2017.

46 See also Phinda family group interview, 8 November 2008; interview with Lali Miya, 2 October 2008.

47 R. Dixon, ‘A Road Haunted by the Past’, *Los Angeles Times*, Los Angeles, 23 May 2009.

48 W. Kalikiti, ‘Road Traffic Accidents and Sudden Death in Zambia: Nature, Causes and Explanations, 1980–2010’ (paper presented at the conference, ‘Managing Uncertainty: Death and Loss in Africa’, University of the Witwatersrand, Johannesburg, 8–10 April 2010).

49 I. Niehaus, ‘Bodies, Heat, and Taboos: Conceptualizing Modern Personhood in the South African Lowveld’, *Ethnology*, 41,3 (June 2002), pp. 189–207. On mapping such moral geographies of death in Mpondoland, see Ka Canham, *Riotous Deathscapes*.

50 Interview with Makhaya Mgangathi, 1 December 2008. Interestingly, this ritual practice was part of the plotline of an episode of the televised drama, *Isidingo*, in 2008.

51 Interview with Khwezi Buyani and Babalo Ncwadi, 20 July 2017.

52 Interview with Welcome Malahle, 12 July 2017; see also Adam Ashforth on the ‘invisibilisation’ of traditional medicine in state discourse in post-apartheid South Africa, and the apparent ‘incommensurability’ of ‘African science’, at least in the public sphere, with bio-medicalised approaches and understandings; A. Ashforth, ‘*Muthi*, Medicine and Witchcraft: Regulating “African Science” in Post-Apartheid South Africa?’, *Social Dynamics*, 31, 2 (2005), pp. 211–42.

53 Interview with Khwezi Buyani and Babalo Ncwadi, 20 July 2017. A large-scale survey by Peltzer and Renner in 2003 showed that male taxi drivers in the Northern Province demonstrated a high level of ‘superstition’ as a ‘distinct attitude’, but that superstition did not correlate with a high level of risk-taking in driving. Although Peltzer and Renner called for more awareness of such ‘non-rational’ attitudes in driving behaviour, their study was not able to hypothesise whether or how such demonstrations of ‘cultural influence’ could be relevant to road safety conceptions or campaigns: K. Peltzer and W. Renner, ‘Superstition, Risk-Taking and Risk Perception of Accidents Among South African Taxi Drivers’, *Accident Analysis and Prevention*, 35, 4 (2003), pp. 619–23.

54 Focus group interview, Khayelitsha township, 11 July 2017; interview with Mthetho Sibaya, 25 July 2017; interview with Wayne Mitten, 21 July 2017; interview with Daniel Beekhof, 14 July 2017.

55 Interview with Apiwe Ginya, 11 July 2019.

56 For an important exception to this trend, focused on the Namibian context, see M. Chatukuta, N. Groce, J. Mindell and M. Kett, ‘Road Traffic Injuries in Namibia: Health Services, Public Health and the Motor Vehicle Accident Fund’, *International Journal of Injury Control and Safety Promotion*, 28, 2 (2021), pp. 167–78. For examples of medico-legal and administrative scholarship on the RAF, see H.W. Hamid and L.D. Naidoo, ‘The Effectiveness of the Road Accident Fund (RAF) Performance-Management Development System at the RAF Office in Durban, South Africa’, *Journal of Public Affairs* 20, 3 (2020), e2106; M. Slabbert and H.J. Edeling. ‘The Road Accident Fund and Serious Injuries: The Narrative Test’, *Potchefstroom Electronic Law Journal/Potchefstroomse Elektroniese Regsblad*, 15, 2 (2012); H. van Biljon, ‘Occupational Therapists in Medico-Legal Work – South African Experiences and Opinions’, *South African Journal of Occupational Therapy*, 43, 2 (August 2013), 27–33.

57 L.A. Ndlovu, ‘An Agenda Setting Analysis: The Application of Kingdon’s Framework to the Road Accident Fund (RAF)’ (MA thesis, University of KwaZulu-Natal, 2008).

58 National Government of South Africa, ‘Road Accident Fund’, available at https://nationalgovernment.co.za/units/view/152/road-accident-fund-raf, retrieved 22 February 2023.

59 The RAF was not the first legislative attempt by the South African state to address road accident claims. The Motor Vehicle Assurance (MVA) Act of 1942 established compulsory third-party insurance. This was later augmented by the Motor Vehicle Accident Fund (1965), which was brought in to re-insure private insurance companies which had been covering MVA claims. Importantly, in 1986 the Motor Vehicle Accident Act introduced a levy on fuel to pay for the compensation of bodily injury, a direct financial precursor of the RAF system. The basis for this funding possibly drew on global north models – in the middle decades of the 20th century in the United States, as Norton points out, a gasoline tax funded federal and state efforts to implement safe highway design. The 1997 RAF Act was developed in part to centralise the administration of the national insurance system and to address weaknesses in the MVA model, in particular the reliance on insurance companies as agents for the MVA; Norton, ‘Four Paradigms’, p. 327.

60 K. Satchwell, ‘Road Accident Fund Commission Report 2002’ (Pretoria, Ministry of Transport, 2002). For a critique of the RAF Amendment Act, see H. Klopper, ‘Some Observations on the RAF Amendment Act’, *De Rebus*, 458 (October 2006), pp. 28–32.

61 J. Seekings and N. Nattrass, *Class, Race, and Inequality in South Africa* (New Haven, Yale University Press, 2005); H. MacGregor, ‘“The Grant is What I Eat”: The Politics of Social Security and Disability in the Post-Apartheid South African State’, *Journal of Biosocial Science*, 38, 1 (January 2006), pp. 43–55. For early iterations of this scholarship, see R. Naidoo, ‘Pensions and Investment, Retirement Funds and the Dismantling of Economic Apartheid’, in J. Baskin (ed.), *Against the Current: Labour and Economic Policy in South Africa* (Johannesburg, Ravan Press, 1996); S. van der Berg, ‘South African Social Security under Apartheid and Beyond’, *Development Southern Africa*, 14, 4 (1997), pp. 481–503.

62 E. Torkelson, ‘Deserving and Undeserving Welfare States: Cash Transfers and Hegemonic Struggles in South Africa’, *Journal of Southern African Studies*, 48, 1 (2022), pp. 43–60.

63 Interview with Katie Hamilton, 21 July 2017; notes from discussion with Claire Penn, 26 August 2017; interview with Minah Koela, 3 July 2017; interview with Lulama Mthimkhulu, 12 July 2019.

64 See E. Moore and J. Seekings, ‘Consequences of Social Protection on Intergenerational Relationships in South Africa: Introduction’, *Critical Social Policy*, 39, 4 (2019), pp. 513–24; R. Lee, *African Women and Apartheid: Migration and Settlement in Urban South Africa* (London, I.B. Tauris, 2009); A.D. Spiegel, ‘Reconfiguring the Culture of Kinship: Poor People’s Tactics during South Africa’s Transition from Apartheid’, *Africa: The Journal of the International African Institute*, 88, S1 (2018), pp. S90–S116.

65 S.J. Cooper-Knock and A. Macdonald, ‘A Summons to the Magistrates Courts in South Africa and Uganda’, *African Affairs*, 119, 477 (October 2020), pp. 552–77.

66 M. Bolt, ‘Homeownership, Legal Administration, and the Uncertainties of Inheritance in South Africa’s Townships: Apartheid’s Legal Shadows’, *African Affairs*, 120, 479 (2021), pp. 219–41.; M. Bolt, ‘Fluctuating Formality: Homeownership, Inheritance, and the Official Economy in Urban South Africa’, *Journal of the Royal Anthropological Institute*, 27, 4 (December 2021), pp. 976–94.

67 My research assistant unwittingly encountered this sobering realisation in the process of attempting to claim from the RAF to cover the considerable funeral expenses incurred burying her sister, who was struck down by a car in Khayelitsha in 2016 and eventually died, four months later, from her injuries. Interview with Minah Koela, 3 July 2017.

68 E. Pettigrew, *Invoking the Invisible in the Sahara: Islam, Spiritual Mediation, and Social Change* (Cambridge, Cambridge University Press, 2023).

69 For a bioethical approach to road safety, see L. Randall, ‘“Coffins on Wheels”: A Bioethical Study of Work Conditions, Driver Behaviour and Road Safety in the Johannesburg Minibus Taxi Industry’ (PhD thesis, University of the Witwatersrand, 2019).

